# Redox Cycling Dioxonaphthoimidazoliums Disrupt Iron Homeostasis in Mycobacterium bovis Bacillus Calmette-Guérin

**DOI:** 10.1128/spectrum.01970-22

**Published:** 2022-11-15

**Authors:** Ming Li, Yoshiyuki Yamada, G. Marcela Rodriguez, Thomas Dick, Mei Lin Go

**Affiliations:** a Department of Pharmacy, Faculty of Science, National University of Singapore, Singapore, Singapore; b Department of Immunology and Microbiology, Yong Loo Lin School of Medicine, National University of Singapore, Singapore, Singapore; c The Public Health Research Institute at New Jersey Medical School, Rutgers, The State University of New Jersey, Newark, New Jersey, USA; d Center for Discovery and Innovation, Hackensack Meridian Health & Department of Medical Sciences, Hackensack Meridian School of Medicine, Nutley, New Jersey, USA; e Department of Microbiology and Immunology, Georgetown University, Washington, DC, USA; University of North Carolina at Chapel Hill

**Keywords:** *Mycobacterium bovis* BCG, ROS, redox cycling, iron homeostasis

## Abstract

The dioxonaphthoimidazolium scaffold is a novel, highly bactericidal redox cycling antituberculosis chemotype that is reliant on the respiratory enzyme Type II NADH dehydrogenase (NDH2) for the generation of reactive oxygen species (ROS). Here, we employed Mycobacterium bovis Bacillus Calmette-Guérin (M. bovis BCG) reporter strains to show that ROS generated by the redox cycler SA23 simulated an iron deficient state in the bacteria, which led to a compensatory increase in the expression of the iron acquisition *mbtB* gene while collaterally reducing the expression of the iron storage *bfrB* gene. Exacerbating the iron deficiency via the inclusion of an iron chelator or aggravating oxidative stress by deploying a catalase (KatG) loss-of-function mutant strain enhanced the activity of SA23, whereas a combined approach of treating the *katG* mutant strain with an iron chelator led to even greater gains in activity. Our results support the notion that the activity of SA23 pivots on a vicious cycle of events that involve the derailment of iron homeostasis toward greater acquisition of the metal, overwhelmed oxidative stress defenses due to enhanced Fenton reactivity, and, ultimately, self-inflicted death. Hence, we posit that redox cyclers that concurrently perturb the iron equilibrium and cellular respiration are well-positioned to be potent next-generation anti-tubercular drugs.

**IMPORTANCE** Cellular respiration in mycobacteria is a potentially rich target space for the discovery of novel drug entities. Here, we show that a redox cycling bactericidal small molecule that selectively activates a respiratory complex in mycobacteria has the surprising effect of disrupting iron homeostasis. Our results support the notion that the disruption of cellular respiration is a potent driver of reactive oxygen species (ROS) generation by the redox cycling molecule. Mycobacteria respond by acquiring iron to restore the levels depleted by the prevailing oxidizing conditions, which inadvertently trigger the compensatory acquisition of the metal. This leads to overwhelmed oxidative stress defenses and yet more iron depletion. For organisms that are unable to break out of this pernicious cycle of events, cell death is the inevitable outcome. Hence, aberrant ROS production by a redox cycling bactericidal agent inflicts a plethora of damaging effects on mycobacteria, including the derailment of iron homeostasis.

## INTRODUCTION

**FIG 1:**
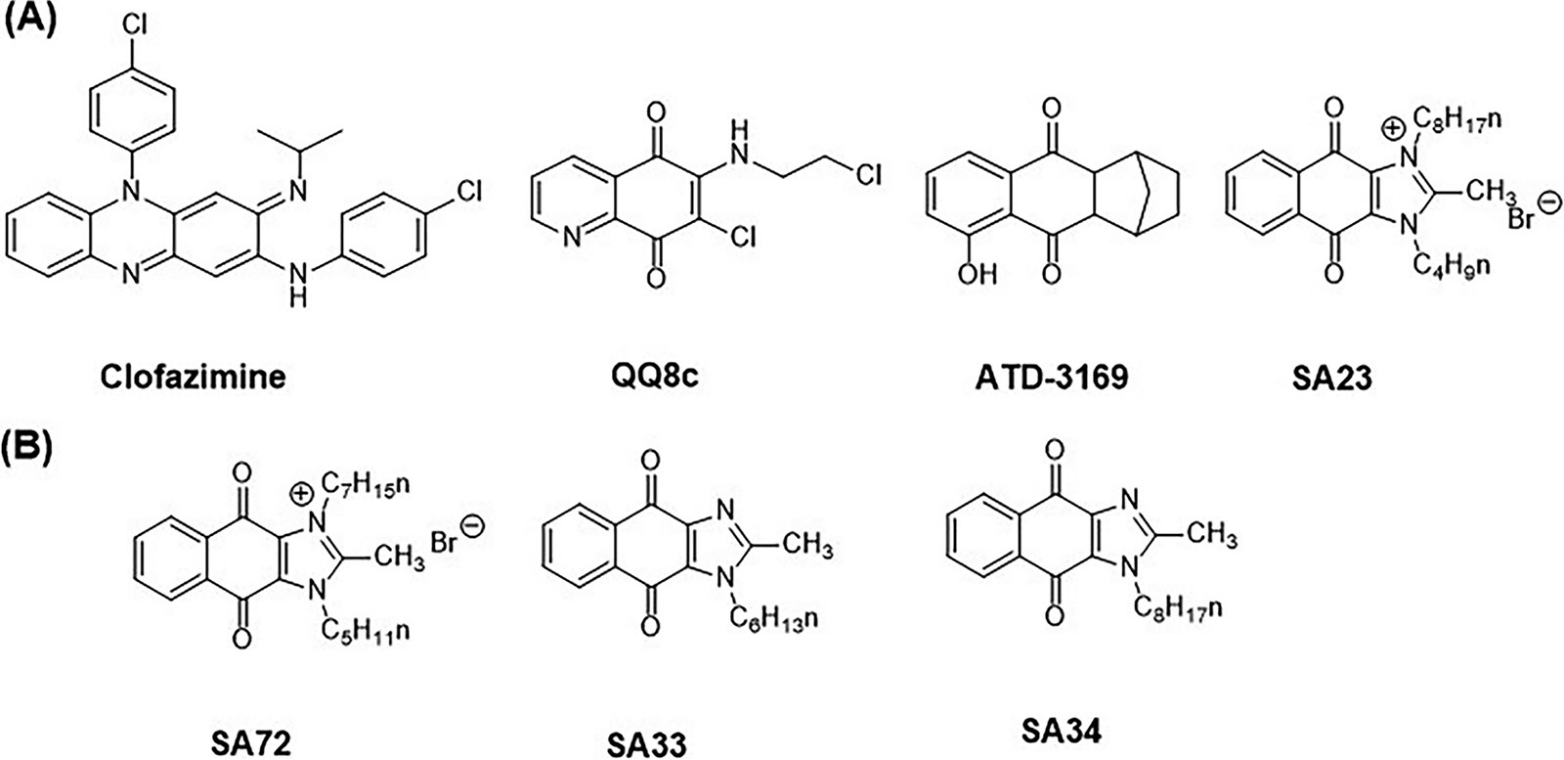
(A) Structures of representative redox cycling antimycobacterial agents. The systemic nomenclature of SA23 is 3-butyl-2-methyl-1-octyl-4,9-dioxo-4,9-dihydro-1*H*-naphtho[2,3-*d*]imidazol-3-ium bromide. Minimum inhibitory concentrations (MIC_90_) on *M. tb* H37Rv or M. bovis BCG are (i) Clofazimine MIC_MtbH37Rv_, 0.25 μM (28); MIC_BCG_, 0.4 μM (11); (ii) QQ8c MIC_MtbH37Rv_, 8.1 μM; MIC_BCG_, 8.1 μM (10); (iii) ATD-3169 MIC_MtbH37Rv_, 3.1 μM (12); (iv) SA23 MIC_MtbH37Rv_, 1.5 μM; MIC_BCG_, 0.26 μM (11). (B) Structures of SA23 analogues: SA72 MIC_MtbH37Rv_, 1.2 μM; MIC_BCG_, 0.27 μM; SA33 MIC_BCG_, 15 μM; SA34 MIC_BCG_, 11 μM (11).

Tuberculosis has caused more deaths than any other infectious disease attributed to a single pathogen, surpassing human immunodeficiency virus/acquired immunodeficiency syndrome (HIV/AIDS) and malaria, and it was overtaken only by coronavirus disease 2019 (COVID-19) in 2020 (1). The eradication of tuberculosis has proved daunting, largely due to the unique ability of the causative organism Mycobacterium tuberculosis (*M. tb*) to exist in a metabolically active replicating state that succumbs to drug treatment (in the absence of resistance) and in a nonreplicating dormant state that is phenotypically drug-tolerant. In the search for novel druggable targets against tuberculosis, mycobacterial bioenergetics has emerged as an incipient target space for drug discovery (2–4). Several broad strategies have been proposed for future drug development, namely, the inhibition of respiratory chain components (5), the inhibition of ATP synthesis (6, 7), the dissipation of the electrochemical gradient driving the electron transport chain (8), and the activation of respiratory complexes (9, 10). The activation of the mycobacterial respiratory enzyme Type II NADH dehydrogenase (NDH2) has been reported for several redox cycling antibacterial agents, notably clofazimine, the quinolinequinone QQ8c, and the dioxonaphthoimidazolium SA23 (9–11) (Fig. 1A). Briefly, these agents increase oxygen consumption by stimulating the oxidation of NADH by NDH2, which is the primary entry point of electrons into the mycobacterial electron transport chain. The electrons from NADH are then diverted from menaquinone, which is the endogenous substrate of NDH2, toward the redox cycler, thereby leading to an increase in the turnover of ROS. Hence, the activation of NDH2 is pivotal to augmenting the redox cycling activity of these compounds and is causal to their bactericidal activity (11). NDH2 may play a related role in the antibacterial activity of the hexahydroanthracenedione redox cycler ATD-3196 (12) (Fig. 1A), but this has yet to be established. Interestingly, ATD-3196 was reported to induce the transcription of the mycobacterial genes involved in the oxidative stress response (*furA*), iron homeostasis (*ideR*), and iron storage (*bfrB*) (12). This raises the question as to whether the disruption of iron homeostasis in mycobacteria would extend to other redox cyclers, in particular, SA23, which is one of the more potent investigational, redox-guided antibacterials that has been reported to date (11).

Mycobacterial defenses against oxidative stress are adapted to the iron environment, and there is a causal connection between cellular levels of iron and mycobacterial resistance to oxidative stress (13–16). In the presence of readily oxidizable ferrous ions (Fe^2+^), the transcription factors FurA and IdeR in mycobacteria bind to specific DNA sequences (iron boxes) in the promoter regions of the genes that they regulate, thereby controlling transcription (17–19). Consequently, both proteins are highly sensitive to the presence of ROS, presumably due to the oxidative loss of Fe^2+^ or the altered binding affinity of the oxidized protein (13).

**FIG 2:**
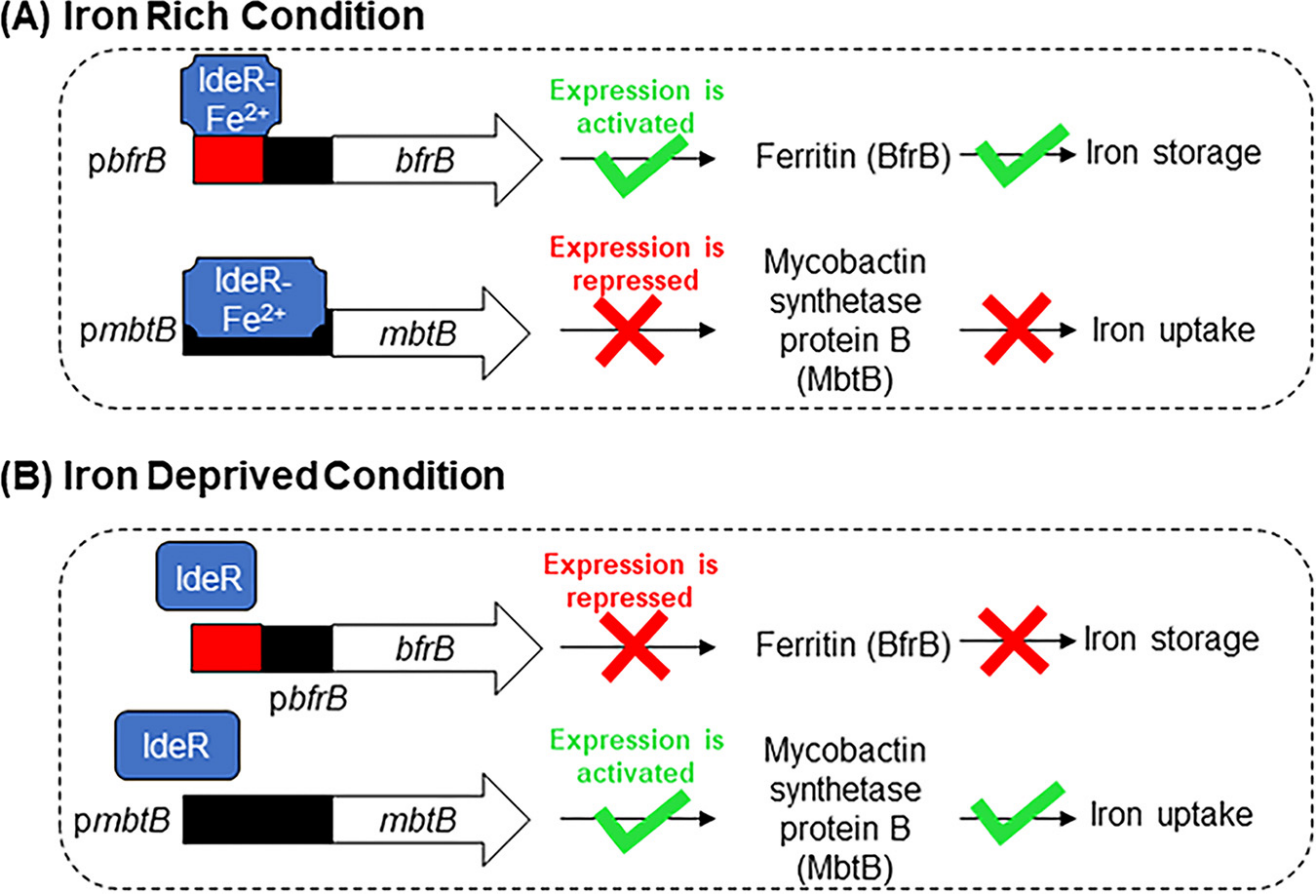
(A) Under iron rich conditions, IdeR binds to multiple iron boxes (red) located upstream of the *bfrB* promoter (black) (29) and activates the transcription of *bfrB*, which is the gene encoding the major iron storage protein of mycobacteria. In contrast, IdeR binds to the promoter of *mbtB*, which is an iron acquisition gene in the siderophore synthesis cluster and serves to repress transcription. (B) Under iron deprived conditions, IdeR does not bind to the iron box(es) upstream or within the promoter region due to the diminished Fe^2+^ levels. The reverse is now observed, and the transcription of the iron storage gene *bfrB* is repressed, whereas the siderophore synthesis gene *mbtB* is actively transcribed.

The transcription factor IdeR is the major iron regulatory protein in mycobacteria (18, 19). Its regulatory activity is predicated on the repression of iron acquisition genes and the activation of iron storage genes when iron levels are elevated (19) (Fig. 2A). The reverse is observed when iron levels are low (Fig. 2B). In this way, intracellular iron levels are carefully balanced to avoid an excess, which would amplify ROS production and toxicity through the Fenton reaction (Fe^2+^ + H_2_O_2_ → Fe^3+^ + OH• + OH^−^), or a deficit, which, paradoxically, would also precipitate oxidative stress by restricting the availability of iron-containing protective enzymes and defensive oxidative stress proteins (20, 21).

Fur proteins (FurA, ZurB) are transcriptional repressors that exhibit Fe^2+^-dependent DNA binding activity and regulate several genes involved in various functions, such as the oxidative stress response and the metabolism of iron (16, 22). FurA negatively controls its own transcription by binding to the p*furA* promoter in a redox-dependent manner (17). Hence, FurA binds to its promoter p*furA* under reducing conditions to repress transcription; however, when it is exposed to oxidative stress, its binding affinity is diminished, and genes (*furA*, *katG*) that are normally repressed by FurA are upregulated (17). Indeed, we observed that the oxidative stress induced by SA23 increased the transcriptional activity of p*furA* (11), which led us to posit that SA23 is likely to affect the expression of genes involved in iron storage and acquisition, as in ATD-3196 (12). This notion was further reinforced by a report that a *bfrB* gene deletion mutant of *M. tb* was unusually sensitive to oxidative stress caused by hydrogen peroxide (H_2_O_2_) and the redox cycler menadione (15). This enhanced sensitivity was annulled when the mutant strain was pretreated with an iron chelator, suggesting that reducing the iron overload induced by the deletion of *bfrB* diminished the susceptibility of the mutant strain to oxidative stress. Relatedly, oxidative stress induced by the redox cycler ATD-3196 led to the upregulation of the iron storage gene (*bfrB*) in mycobacteria, possibly as a compensatory response to the elevated iron levels caused by the leaching of the metal from iron-containing proteins (12). Interestingly, the response of mycobacteria to low levels of exogenously added H_2_O_2_ was to upregulate the siderophore synthesis genes (*mbt*) (13, 14). The fact that an oxidant (H_2_O_2_) should increase iron uptake may seem counterintuitive, as elevated iron levels would promote hydroxyl radical formation through the Fenton reaction and exacerbate the oxidative stress already imposed by H_2_O_2_. Presumably, the increase in iron uptake was necessary to restore the iron levels in critical proteins that were depleted by the oxidative loss of Fe^2+^. Clearly, the intracellular iron poise is pivotal to mycobacterial defenses against oxidative stress, but it is unclear how cellular iron levels would be altered by the continual generation of ROS by redox cyclers, such as SA23. Furthermore, the possibility that the interplay between iron and ROS levels could blunt the antibacterial activity of SA23 cannot be discounted.

To explore these questions, we interrogated the effects of SA23 on three iron-reporter M. bovis BCG strains in which the expression of red fluorescent protein (RFP) is controlled by promoters that regulate genes involved in iron regulation (*ideR*), acquisition (*mbtB*), and storage (*bfrB*). Also investigated were three analogues of SA23, namely, SA72, which is comparable to SA23 in terms of its antibacterial activity and mode of action, as well as two less potent and significantly weaker ROS generators, SA33 and SA34 (Fig. 1B) (11).

## RESULTS AND DISCUSSION

Briefly, our protocol involved treating the iron reporter M. bovis BCG strains with various concentrations of the test compounds for 24 h in iron-depleted and iron-supplemented (10 μM) minimal media. The promoter activity of the reporter strain was monitored by RFP fluorescence with corrections made for variations in cell numbers (OD_600_, the optical density at a wavelength of 600 nm). An increase in the fluorescence signal would indicate that a gene is upregulated.

**FIG 3:**
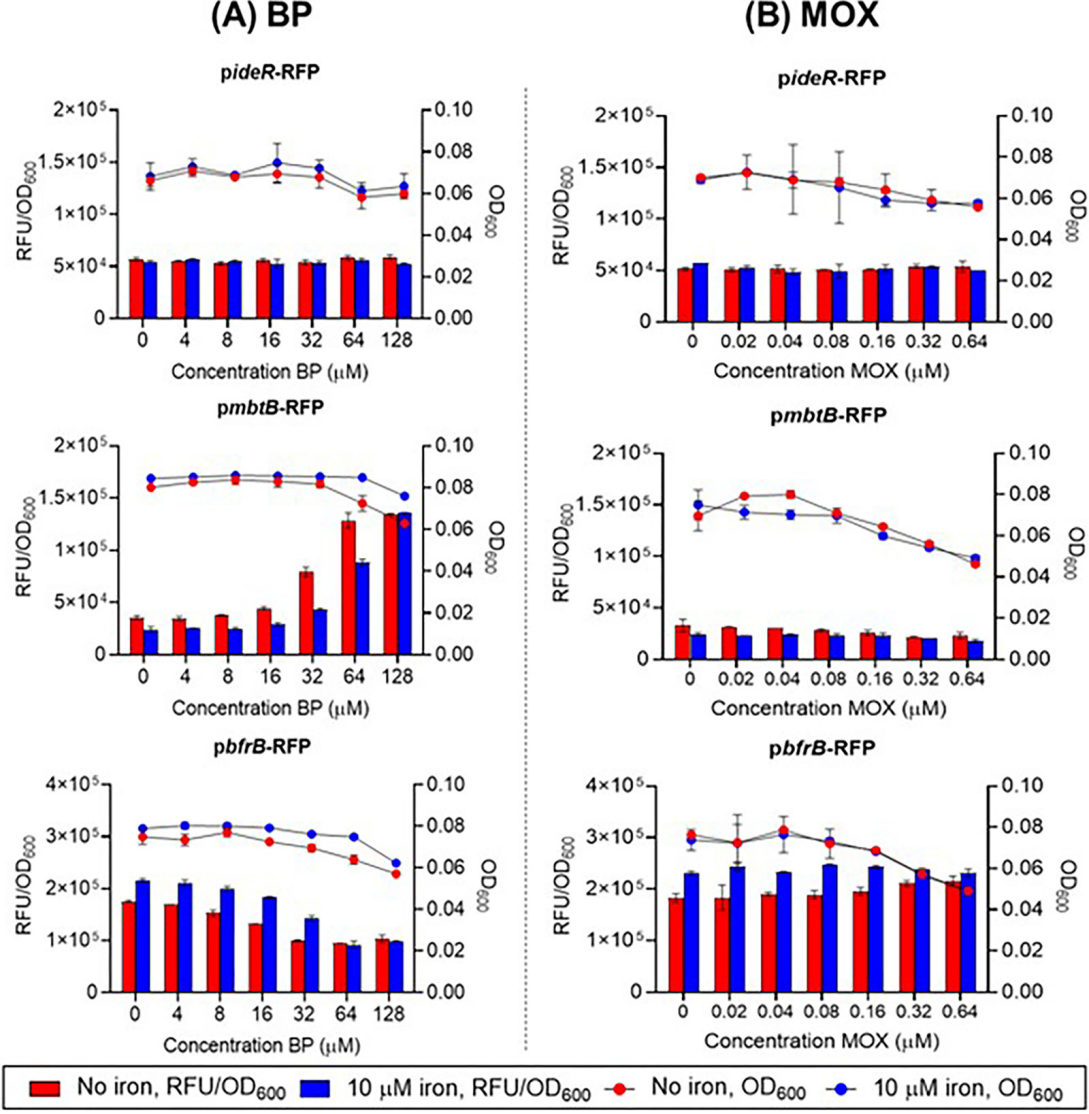
Dose dependent promoter activities of recombinant strains of M. bovis BCG-p*ideR-*RFP, M. bovis BCG-p*mbtB*-RFP, and M. bovis BCG-p*bfrB*-RFP treated with (A) iron chelator 2,2′ bipyridyl (BP) and (B) Moxifloxacin (MOX, negative control) for 24 h in iron-depleted and iron supplemented (10 μM) minimal media. Fluorescence signals (RFU) were normalized against the OD_600_ values of treated cultures to correct for changes in cell numbers. Three independent biological replicates were carried out for each experiment. Similar trends were observed among the replicates. Representative results from one biological replicate with two technical replicates are shown here. The data shown are mean values with standard deviations (SDs) depicted as error bars. Blue and red dots represent OD_600_ readings from iron-depleted and iron-supplemented media, respectively. Blue and red bars represent RFU/OD_600_ readings from iron-depleted and iron-supplemented media, respectively. Panel A shows that the iron chelator BP neither induced nor suppressed the iron regulator gene *(ideR*) in either medium but induced the iron acquisition gene *mbtB* (iron-depleted minimal medium > iron-supplemented minimal medium) and suppressed the iron storage gene *bfrB* (iron-supplemented minimal medium > iron-depleted minimal medium). Panel B shows that MOX did not affect the transcriptional activities of *ideR*, *mbtB*, or *bfrB* in either medium. The dose related OD_600_ signals were constant in both media, except for some losses at the higher test concentrations of MOX on the M. bovis BCG-p*mbtB*-RFP and M. bovis BCG-p*bfrB*-RFP strains (~2-fold decrease in OD_600_).

First, we validated the integrity of the reporter strains by confirming their transcriptional responses to the iron chelator 2,2′-bipyridyl (BP), which simulates low iron conditions (15), and the antibacterial moxifloxacin (MOX), which does not perturb intracellular iron levels (23). As shown in Fig. 3A, the fluorescence emitted by cultures of the p*ideR*-RFP reporter strain that was exposed to the iron chelator BP in both iron-depleted and iron-supplemented media remained constant over the dose range of BP that was explored. Signals from both media were of similar magnitude. Thus, we inferred that the transcription of p*ideR* was unaffected by variations in iron content and that the IdeR protein was available for binding to the iron boxes of the genes under its control. Notwithstanding its availability, IdeR would not be activated for binding to the iron boxes due to the iron deficient state induced by BP. Consequently, genes normally repressed by IdeR (such as *mbt*B for iron acquisition) would be activated, whereas genes induced by IdeR (notably *bfr*B for iron storage) would be repressed. These were indeed evident from the responses of the reporter strains p*mbtB*-RFP and p*bfrB*-RFP (Fig. 3A). Consistent with our interpretation, the transcription of the iron acquisition gene *mbtB* was upregulated as opposed to the transcription of the iron storage gene *bfrB*, which was downregulated. While this dose-dependent trend was evident in both the iron-free and the iron supplemented media, the fluorescence of p*mbtB*-RFP was consistently higher in the iron-depleted media, whereas the fluorescence of the p*bfrB*-RFP cultures was stronger in the iron-supplemented media. These differential responses were in keeping with the iron deficient state induced by BP, which would arguably be more pronounced in the iron-depleted medium versus the iron-supplemented medium. Consequently, iron acquisition would be prioritized in the iron-depleted medium, whereas iron storage would be more pronounced in the iron-supplemented medium.

Next, we investigated the responses of the reporter strains to MOX, which does not affect mycobacterial iron levels (23). Accordingly, MOX neither induced nor suppressed the promoter activities of the reporter strains (*ideR*, *mbtB*, *bfrB*) in the iron-depleted and iron-supplemented media (Fig. 3B). Taken together, the responses elicited from BP and MOX validated our assay protocol and affirmed the integrity of the reporter strains.

**FIG 4:**
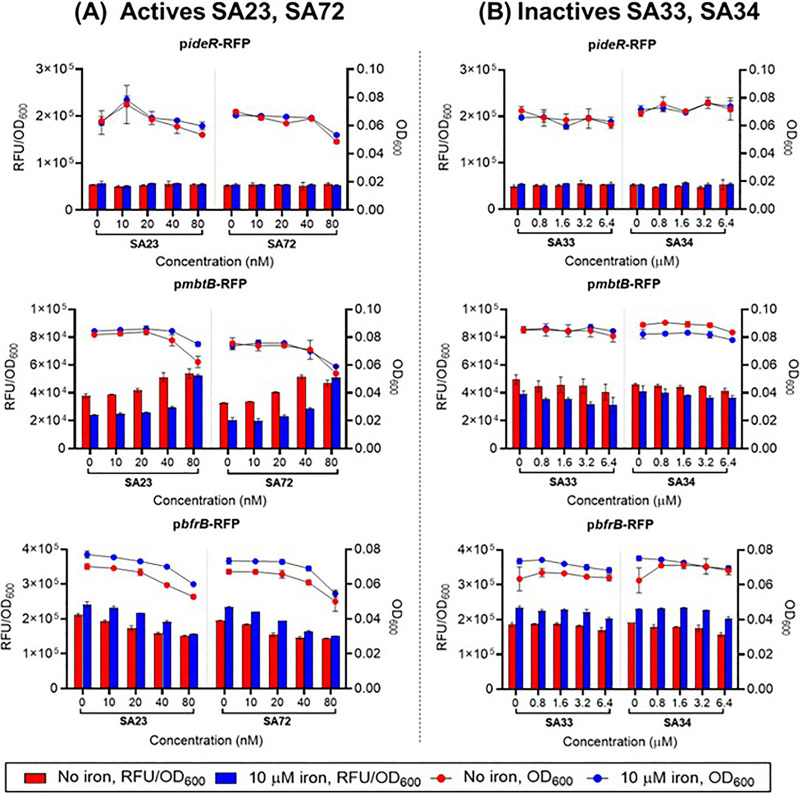
Dose dependent transcriptional activities of iron reporter M. bovis BCG strains treated with (A) “actives” SA23 and SA72 as well as (B) “inactives” SA33 and SA34. Three independent biological replicates were carried out for each experiment. Trends were consistent across these determinations. Representative results from one biological replicate (from two technical replicates) are shown. The data shown are mean values with the SDs depicted as error bars. Dots and bars have the same meanings as in Fig. 3. Panel A shows that the “actives” SA23 and SA72 neither induced nor suppressed the iron regulator gene *(ideR*) in either medium but induced the iron acquisition gene *mbtB* (iron-depleted minimal medium > iron-supplemented minimal medium) and suppressed the iron storage gene *bfrB* (iron-supplemented minimal medium > iron-depleted minimal medium). Panel B shows that the “inactives” SA33 and SA34 did not affect the transcriptional activities of *ideR*, *mbtB*, or *bfrB* in either medium. The recombinant strains retained dose-dependent viabilities in both media.

Second, we investigated the effects of the potent and highly active redox cyclers SA23 and SA72 (“actives”) on the iron reporter strains. Intriguingly, their responses mirrored those of the iron chelator BP. In the iron-depleted and iron-supplemented media, the actives upregulated *mbtB* transcription (increase in iron acquisition), collaterally downregulated *bfrB* transcription (decrease in iron storage), and had no effect on *ideR* (Fig. 4A). We interpreted these responses as being indicative of an iron deficient intracellular milieu that was ostensibly induced by the ROS that were generated by the redox cycling actives. As an initial interaction with Fe^2+^ precedes the binding of IdeR to the iron boxes of relevant genes, the oxidative loss of Fe^2+^ would suppress this interaction and thereby promote the expression of IdeR-repressed genes (*mbtB*) and the repression of IdeR-induced genes (*bfrB*). Thus, the mycobacteria would undergo a phase of increased acquisition and diminished storage of iron.

To confirm the role of ROS in simulating the iron deficient states of SA23/SA72-treated mycobacteria, we compared the effects elicited by these actives with those of the less potent analogues SA33 and SA34, which were also significantly weaker ROS generators (“inactives”) (11). Strikingly, the inactives neither induced nor suppressed the transcriptional activities of *mbtB* and *bfrB* (Fig. 4B). Clearly, no iron deficient state was induced by these compounds, reinforcing the notion that the perturbative effects of the actives on iron homeostasis are related to ROS generation.

Thus far, we have posited that the generation of ROS by the actives induced an iron deficient state in the mycobacteria, which thereby contributed to antibacterial activity. It follows that accentuating the iron deficient state, oxidative stress, or both should augment activity. To this end, we monitored the activity of SA23 in the presence of the iron chelator BP in wild-type (WT) M. bovis BCG and in a *katG* loss-of-function mutant of M. bovis BCG. The presence of BP would aggravate the iron deficient state induced by SA23 in both the WT and *katG* mutant strains, whereas oxidative stress would be more pronounced in the *katG* mutant.

**FIG 5:**
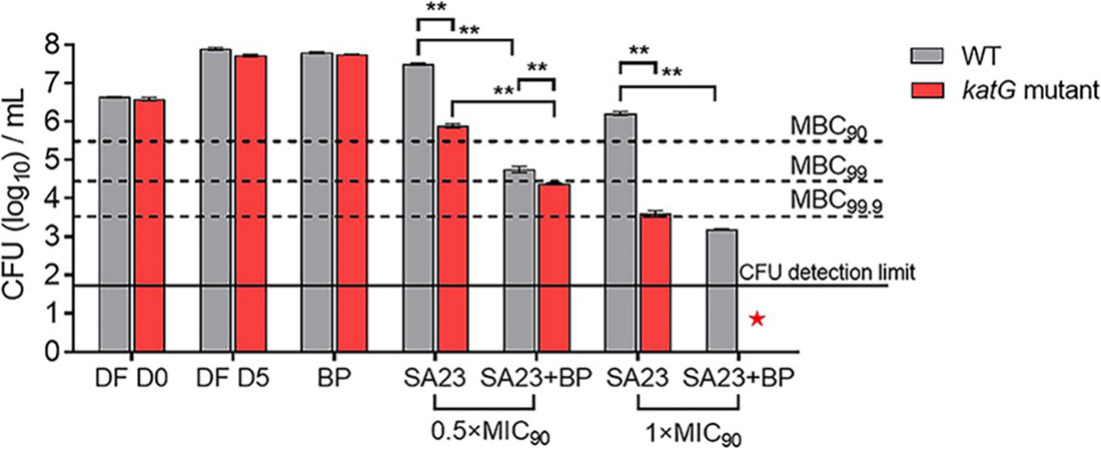
Colony formation in cultures of wild-type (WT) and *katG* loss-of-function mutant M. bovis BCG strains treated with 2,2′-bipyridyl (BP) (80 μM), SA23 (0.16 μM and 0.32 μM, equivalent to 0.5× and 1× the MIC_90_ value, respectively) and combinations of BP and SA23 (SA23+BP) at the stated concentrations. Drug treatments were carried out in catalase free 7H9 broth at 37°C and 110 rpm for 5 days. The aliquots were then withdrawn, serially diluted, and plated out on 7H10 agar for colony-forming unit (CFU) counting. DF D0 and DF D5 were untreated, drug free controls that were monitored at day 0 and day 5 which correspond to the start and end of drug exposure. No significant differences were observed between the gray and red bars at each time point, indicating that the WT and *katG* mutant strains displayed similar growth patterns over the 5-day period. “BP” refers to WT/*katG* mutant cultures treated with BP at 80 μM. The CFU of the “DF D5” and “BP” treatment arms (WT, *katG* mutant) were comparable, indicating that BP was not bactericidal at 80 μM on either strain. In the case of SA23, the cotreatment arm (SA23+BP) was compared to the control arm (SA23) as described in the text. MBC_90_, MBC_99_, and MBC_99.9_ are the concentrations required to reduce the CFU by 10×, 100× and 1,000× compared to DF D0. The solid line represents the approximate limit for CFU detection. The experiment was repeated thrice independently, with one representative set of results shown. **, *P* < 0.01; Student’s *t* test; GraphPad Prism Ver. 8.4.3. The CFU counts are inversely related to antibacterial activity. SA23 exhibited greater activity on the *katG* mutant compared to the WT strain. In the presence of BP, the increase in activity was particularly pronounced in the *katG* mutant strain treated with SA23 at 1× MIC_90_, at which the CFU counts were reduced to a level below the detection limit (indicated by a red star).

Figure 5 shows the antibacterial activities (expressed as colony-forming units [CFU]) of SA23 at two concentrations (0.5× and 1×MIC_90_ which is the minimum inhibitory concentration at which 90% of the isolates are inhibited) on WT M. bovis BCG and on the *katG* mutant in catalase-free media and in BP-supplemented catalase-free media. Two key observations were evident. First, when assessed on the WT cultures, supplementation with BP led to significant dose-dependent increases (reduction in CFU) in the activity of SA23. Enhancement was particularly pronounced at the higher 1× MIC_90_ concentration, at which the addition of BP reduced the CFU to levels below the minimum bactericidal concentration required to kill 99.9% of the bacterial population (MBC_99.9_) of SA23. Second, the augmentation of SA23 activity by BP was even more striking when assessed on the *katG* mutant strain. Previously, we have reported that SA23 was significantly more potent against the *katG* mutant, relative to the WT strain (11). Here, we showed that supplementation with BP further enhanced activity. Notably, the combination of BP and SA23 (1× MIC_90_) successfully reduced the CFU of the mutant strain to levels below the detection limit.

Taken together, these findings corroborated the pivotal roles of iron and ROS in the bactericidal activity of SA23. SA23 induces a state of iron deficiency in the bacteria, as seen from the transcriptional responses of the iron reporter strains (Fig. 4A). Aggravating the iron deficient state (BP supplementation) or oxidative stress (*katG* mutant) potentiated the activity of SA23. When both approaches were concurrently employed, as in the cotreatment of the *katG* mutant with SA23 and BP, the outcome was an impressive enhancement of antibacterial activity.

There are several limitations to the present investigation which could be addressed in follow-up work. Notably, these experiments should be carried out on the human pathogenic *M. tb*. Although the genome of M. bovis BCG exhibits 99.9% identify to that of *M. tb*, several genes present in *M. tb* are not found in M. bovis BCG, which could result in different outcomes (24, 25). It would also be of interest to determine whether the *bfrB* mutant of *M. tb* that was reported by Pandey and Rodriguez (15) is as susceptible to killing by SA23 as it was to antibiotics. A positive finding would have important implications for the design of new therapeutic strategies against *M. tb*. In addition, we have only investigated the bactericidal potency of SA23 against a *katG* mutant strain of M. bovis BCG under conditions of iron deprivation. As a catalase peroxidase, KatG is primarily involved in neutralizing hydrogen peroxide. In the biological milieu, mycobacteria are challenged not only by hydrogen peroxide but also by a plethora of oxidant species (organic hydroperoxides, reactive nitrogen species) (26). A pertinent question would be whether SA23 or other mechanistically related redox cyclers retain the same activity profile against other oxidant species under low iron conditions.

**FIG 6:**
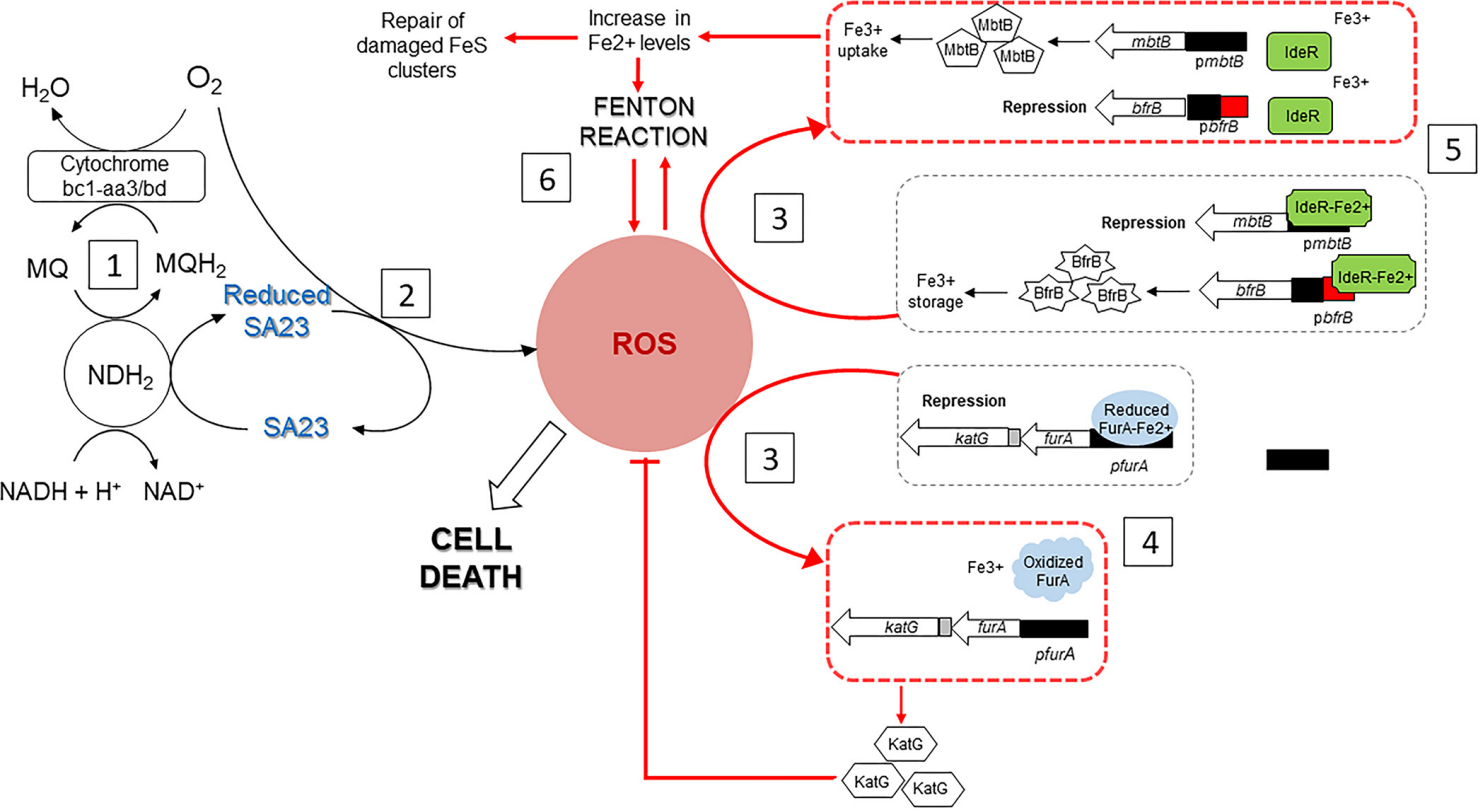
Proposed mechanism of action of SA23 in mycobacteria. (Panel 1) Type II NADH dehydrogenase (NDH2) diverts electrons intended for the reduction of menaquinone (MQ) toward SA23, thus affecting oxidative phosphorylation. (Panel 2) Spontaneous oxidation of reduced SA23 generates reactive oxygen species (ROS). (Panel 3) ROS disrupt iron-containing clusters in enzymes, oxidize Fe^2+^ to the biologically inert Fe^3+^, and/or induce the metal-catalyzed oxidation of the critical regulatory proteins FurA and IdeR. (Panel 4) SA23-induced oxidative stress suppresses the binding of FurA to p*furA*, thereby upregulating the transcription of *katG*, which encodes the antioxidant enzyme KatG. (Panel 5) SA23-induced oxidative stress induces an iron (Fe^2+^)-deficient state, which reduces the binding of IdeR to the promoter sequences (p*bfrB*), thereby suppressing the transcription of the iron storage *bfrB* gene while increasing its interactions with the promoter sequences (p*mbtB*) responsible for the transcription of the iron acquisition *mbtB* gene. (Panel 6) This sets in motion a vicious cycle, wherein redox cycling dysregulates iron levels, increases the likelihood of Fenton reactivity, and hastens mycobacterial cell death.

Figure 6 summarizes our present understanding of the mode of action of SA23. We have shown that the antibacterial activity of SA23 is causally related to the generation of ROS from redox cycling and is dependent on the respiratory enzyme NDH2 to sustain the process (11). Our current findings support the interpretation that an influx of ROS would derail iron homeostasis, tilting it toward an iron deficient state, which would in turn trigger compensatory iron uptake to restore the Fe^2+^ levels depleted by the oxidizing conditions. The inflow of Fe^2+^ would pose a serious challenge to oxidative stress defenses due to an increase in Fenton reactivity, and, for bacteria unable to remediate this situation, self-inflicted death would follow. Hence, the bactericidal activity of SA23 leverages on a pernicious cycle of events arising from disruptions to two interrelated systems governing cellular respiration and iron homeostasis. SA23 activates NADH oxidation via the respiratory enzyme NDH2 but subverts electron flow from the normal adenosine triphosphate (ATP) production pathway to a ROS-generating route that is enabled by the redox cycling activity of SA23. Aberrant ROS production induces a plethora of damaging effects to cellular components (lipids, DNA, proteins), and this is further aggravated by the derailment of iron homeostasis. Taken together, our findings support the notion that SA23 and other mechanistically related redox cyclers are uniquely positioned to be extraordinarily bactericidal next-generation anti-tubercular drugs.

## MATERIALS AND METHODS

### Bacterial strains and culture conditions.

M. bovis BCG Pasteur ATCC 35734 was purchased from the American Type Culture Collection and maintained at 37°C in either complete Middlebrook 7H9 broth (BD Difco, Detroit, MI, USA) supplemented with 0.05% (vol/vol) Tween 80, 0.5% (vol/vol) glycerol, and 10% (vol/vol) Middlebrook albumin-dextrose-catalase or on complete Middlebrook 7H10 agar (BD Difco, Detroit, MI, USA) supplemented with 0.5% (vol/vol) glycerol and 10% (vol/vol) oleic acid-albumin-dextrose-catalase. Catalase-free 7H9 broth was prepared with 0.05% (vol/vol) Tween 80, 0.5% (vol/vol) glycerol, 0.5% (wt/vol) bovine albumin, 0.2% (wt/vol) glucose, and 0.085% (wt/vol) NaCl. A *katG* loss-of-function mutant of M. bovis BCG harboring a GC deletion mutation at 633 to 634 bp was prepared as described (11).

### Preparation of iron-depleted and iron-supplemented minimal media.

Iron-depleted minimal medium was prepared and used for the iron reporter assays (25). The iron-depleted minimal media contained 0.5% (wt/vol) l-asparagine, 0.5% (wt/vol) KH_2_PO_4_, 2% (vol/vol) glycerol, 0.05% (vol/vol) Tween 80, and 10% (vol/vol) Middlebrook albumin-dextrose-catalase. Its pH was adjusted to 6.8 with NaOH. To remove metal ions, the medium was treated with Chelex-100 (Bio-Rad, Hercules, CA, USA) at 5 g/L for 24 h at 4°C with agitation. The Chelex-100 resin was removed by filtration through a 0.22 μm filter (Sigma-Aldrich; Merck Millipore, Darmstadt, Germany). Thereafter, every liter of medium was supplemented with 0.5 mg sterile ZnCl_2_, 0.1 mg MnSO_4_, and 40 mg MgSO_4_. To obtain a minimal medium with 10 μM iron, 167 μL of sterile 60 mM FeCl_3_ solution was added to every liter of medium.

### Generation of iron reporter strains of M. bovis BCG.

To generate the iron reporter strains, transcriptional promoter reporter fusions were generated and inserted into plasmid pMV262 as described previously (27). The primers used for the construction of the reporter plasmids are shown in Table 1. PCR was performed using KOD FX Neo DNA polymerase (TOYOBO, Osaka, Japan), according to the manufacturer’s instructions. For generating electrocompetent M. bovis BCG, bacteria were grown at 37°C in complete 7H9 broth to an OD_600_ value of 0.2. 2 M glycine (0.1 volume) were added, and the cells were further incubated for 16 h, washed 3 times with washing buffer (10% [vol/vol] glycerol and 0.05% [vol/vol] Tween 80 in MilliQ H_2_O), and resuspended in a 0.02 volume of the initial culture. Electrocompetent M. bovis BCG cells were mixed with 100 ng of plasmid, and electroporation was performed with a Gene Pulser apparatus (Bio-Rad, Hercules, CA, USA) at 2,500 V with a capacity of 25 μF, and a resistance of 1,000 Ω. The bacteria were cultured overnight in fresh complete 7H9 broth and plated on 7H10 agar with 25 μg/mL of kanamycin.

**TABLE 1:** Red fluorescence protein (RFP) promoter reporter plasmids and primers used in this study (30)

Plasmid name	Backbone plasmid (digested with)	Inserted promoter (PCR-amplified DNA fragments digested with NotI and BamHI)				Inserted reporter (PCR product digested with BamHI and EcoRI)	
		Upstream of	Size (bp)	Primer name	Primer sequence	Primer name	Primer sequence
P-bfrB-RFP	pMV262 (NotI–EcoRI)	BCG_3904	200	P-bfrB-F(NotI)	gcggccgcTAACGACACGCTGCCGAATAT	mCh-F(BamHI)	ccgggatccATGGTGAGCAAGGGCGAGG
				P-bfrB-R(BamHI)	ccgggatccAAAATGCAGATTAACGCAGGTAA	mCh-R(EcoRI)	ccggaattcCTACTTGTACAGCTCGTCCAT
P-mbtB-RFP	pMV262 (NotI–EcoRI)	BCG_2397c	242	P-mbtB-F(NotI)	gcggccgcATCGGAGAGCACGGTGTCCAGG	mCh-F(BamHI)	ccgggatccATGGTGAGCAAGGGCGAGG
				P-mbtB-R(BamHI)	ccgggatccAAACCCTCCCCTGTTAGCACAG	mCh-R(EcoRI)	ccggaattcCTACTTGTACAGCTCGTCCAT
P-ideR-RFP	pMV262 (NotI–EcoRI)	BCG_2724	213	P-ideR-F(NotI)	gcggccgcGTTCGTCAGATCGAGCGCGACG	mCh-F(BamHI)	ccgggatccATGGTGAGCAAGGGCGAGG
				P-ideR-R(BamHI)	ccgggatccTCAGCACCCTCCATTGCAGCTGA	mCh-R(EcoRI)	ccggaattcCTACTTGTACAGCTCGTCCAT

### Iron reporter assay.

The iron reporter assay was carried out with dual read-outs of absorbance (OD_600_) and relative fluorescent units (RFU, Ex/Em 587 nm/630 nm) on an Infinite M200 Pro plate reader (Tecan). Briefly, mid-log-phase (OD_600_ value of 0.4 to 0.6) M. bovis BCG in minimal medium that was supplemented with 10 μM iron (FeCl_3_) were spun down at 3,200 × *g* for 10 min, washed once with an iron-depleted minimal medium, and adjusted to an OD_600_ value of 0.2 in fresh iron-depleted minimal medium. The cell suspension (100 μL) was inoculated into flat-bottomed, transparent 96-well plates (Costar, Corning Incorporated, Corning, NY, USA) that contained equal volumes (100 μL) of drug gradients in iron-depleted minimal medium. The microplates were then sealed with Breathe-Easy membranes (Sigma-Aldrich, Burlington, MA, USA) and incubated at 37°C and 110 rpm for 24 h of incubation. Thereafter, the OD_600_ and RFU values were recorded, and the RFU value was normalized against the OD_600_ value for final presentation. To test the responses of the iron reporter strains in the presence of iron, the assay was repeated using cell suspensions prepared in an iron-supplemented (10 μM) minimal medium. Three independent biological replicates were carried out, and representative results from one biological replicate with two technical replicates are shown in Fig. 4.

### Bactericidal potentiating effects of iron chelator 2,2′-bipyridyl (BP).

To determine the potentiating effects of 2,2′-bipyridyl (BP) (Sigma-Aldrich, Burlington, MA, USA) on the bactericidal activity of SA23, mid-log-phase wild-type and *katG* loss-of-function mutant (11) M. bovis BCG cultures were diluted at an OD_600_ value of 0.05 in catalase-free complete 7H9 broth. The cell suspensions were then treated with test compound SA23 at 0.5× and 1× MIC_90_ at 37°C and 110 rpm for 5 days with or without the presence of 80 μM BP. After 5 days of drug treatment, aliquots of the cultures were serially diluted and plated on complete 7H10 agar for CFU counting. Each experiment was independently repeated thrice, with one representative set of results (containing two technical replicates) being shown in Fig. 3.
